# HCMV miRNA Targets Reveal Important Cellular Pathways for Viral Replication, Latency, and Reactivation

**DOI:** 10.3390/ncrna4040029

**Published:** 2018-10-22

**Authors:** Nicole L. Diggins, Meaghan H. Hancock

**Affiliations:** Vaccine and Gene Therapy Institute, Oregon Health and Science University, Beaverton, OR 97006, USA; diggins@ohsu.edu

**Keywords:** cytomegalovirus, miRNA, latency

## Abstract

It is now well appreciated that microRNAs (miRNAs) play a critical role in the lifecycles of many herpes viruses. The human cytomegalovirus (HCMV) replication cycle varies significantly depending on the cell type infected, with lytic replication occurring in fully-differentiated cells such as fibroblasts, endothelial cells, or macrophages, and latent infection occurring in less-differentiated CD14+ monocytes and CD34+ hematopoietic progenitor cells where viral gene expression is severely diminished and progeny virus is not produced. Given their non-immunogenic nature and their capacity to target numerous cellular and viral transcripts, miRNAs represent a particularly advantageous means for HCMV to manipulate viral gene expression and cellular signaling pathways during lytic and latent infection. This review will focus on our current knowledge of HCMV miRNA viral and cellular targets, and discuss their importance in lytic and latent infection, highlight the challenges of studying HCMV miRNAs, and describe how viral miRNAs can help us to better understand the cellular processes involved in HCMV latency.

## 1. Introduction

Human cytomegalovirus (HCMV) is a beta-herpesvirus commonly found in the human population [1], with an estimated seroprevalence of 40 to greater than 90% worldwide, depending on location and socioeconomic status [2]. Successful infection of the host is critically dependent on the ability of HCMV to establish a latent infection, undergo periodic reactivation events, and evade the host immune response. Although HCMV infection is generally asymptomatic, congenital infection with HCMV can lead to serious birth defects including hearing loss, cognitive impairment, and microcephaly [3]. Moreover, reactivation of HCMV causes significant disease in immunocompromised patients such as human immunodeficiency virus (HIV)-infected individuals or those undergoing immunosuppressive cancer therapies, and remains a significant cause of morbidity and mortality in solid organ and allogeneic hematopoietic stem cell transplant recipients [4,5].

Human cytomegalovirus is an enveloped virus containing a ~230 kB double-stranded DNA (dsDNA) linear genome encoding at least 170 proteins, as well as multiple small and long non-coding RNAs, making HCMV the largest of the herpes viruses [6,7]. A temporally controlled cascade of gene expression occurs following viral entry and translocation of the genome to the nucleus. This is classified into three major phases: immediate early (IE), early (E), and late (L) gene expression [8,9,10]. IE genes are transcribed from the major immediate early promoter (MIEP) independently of de novo viral protein synthesis. IE1 and IE2 play essential roles in evasion of the intrinsic and innate immune responses, as well as in viral replication as transcription factors necessary for early gene expression. Early genes are involved in viral DNA replication, and in turn, activate late gene expression important for capsid assembly and virion release [1,11,12]. Newly-formed capsids are then trafficked to the virion assembly compartment (VAC) for maturation and envelopment. The VAC is a perinuclear cytoplasmic compartment containing viral tegument and envelope proteins, and is formed during the late phase of infection by remodeling multiple cellular components, including the endoplasmic reticulum-Golgi intermediate compartment (ER-GIC), the trans-Golgi network (TGN), and early and late endosomes [13,14,15]. Transit of the virus from the VAC to the cell surface occurs via the secretory pathway in an incompletely understood process [1,13,16]. 

Most organs and tissues can be infected with HCMV in vivo due to the broad cell tropism of the virus, and the outcome of infection is largely cell type-dependent. For example, fibroblasts and smooth muscle cells are fully permissive to lytic replication resulting in genome amplification and release of progeny virus particles, contributing to the dissemination and pathogenesis of the virus [17]. HCMV also infects the endothelial and epithelial cells of many organs, and undergoes a more protracted replication cycle that results in persistent low-level release of virus. Latent infection occurs in less differentiated CD34+ hematopoietic progenitor cells (HPCs) in the bone marrow as well as CD14+ monocytes [18,19,20]. After viral entry into these cell types, a short burst of viral gene expression has been noted in in vitro systems, and is followed by a latent state marked by the presence of viral genome but limited gene expression and the absence of newly formed virions. Latency necessarily requires a careful balance between host and virus, whereby the viral genome is maintained, but pathogenesis is limited to avoid immune clearance of infected cells. The limited viral gene expression that occurs during latency implies important roles for the gene products in latency establishment and maintenance, as well as in sensing signals for viral reactivation [18,21]. For successful reactivation from latency, the HCMV genome must remain poised to resume full viral gene expression under the correct conditions, such as following mobilization of stem cells from the bone marrow [22,23] and myeloid differentiation into macrophages [24,25]. Reactivation of the latent virus is key to seeding new sites of infection, and in immunocompetent individuals is controlled by a robust T-cell mediated immune response [18,26]. 

Given that HCMV can establish distinct gene expression patterns depending on the cell type infected, the virus must carefully manipulate the host cell environment as well as its own gene expression in order to achieve different life cycles. One way that protein abundance can be modulated by HCMV is through the expression of viral microRNAs (miRNAs). miRNAs are short ~22 nucleotide non-coding RNAs that are incorporated into the multi-protein RNA-induced silencing complex (RISC), and target the RISC complex to messenger RNAs (mRNAs) through partially-complementary sequences generally found in the 3’ untranslated region (3’ UTR) and nucleotides 2–8 (the ‘seed sequence’) of the miRNAs. Interaction between the RISC complex and the target mRNA results in translational repression, by inhibiting translation initiation or elongation factors, and/or mRNA decay, through the recruitment of deadenylation proteins [27,28,29].

**Figure 1:**
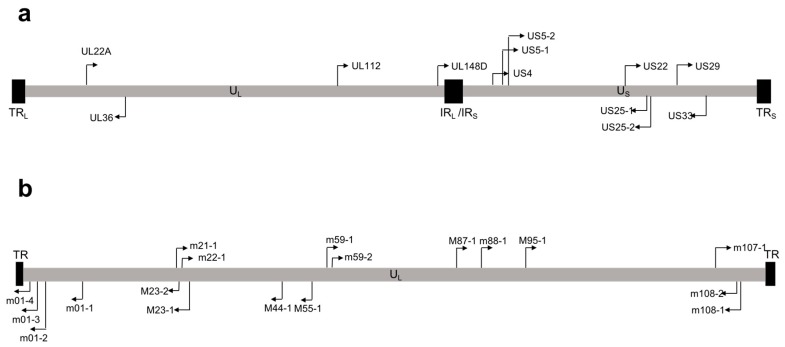
Map of microRNAs (miRNAs) encoded by (**a**) human cytomegalovirus (HCMV) and (**b**) mouse cytomegalovirus (MCMV). Location of HCMV pre-miRNAs are shown on the genome. Black arrows indicate orientation on the genome. TR_L/S_, tandem repeat long/short; U_L/S_, unique long/short; IR_L_/IR_S_, internal repeat long/short.

Over 250 viral miRNAs have been identified, with the vast majority encoded by the herpes virus family [30]. miRNAs encoded by HCMV were first identified by Pfeffer et al. [31], who predicted and cloned nine HCMV pre-miRNAs. Subsequent studies by two independent groups confirmed expression of these miRNAs during viral infection by Northern blot [32,33], and additionally identified miR-US4 [33]. Later deep sequencing studies of lytically-infected human fibroblasts detected a total of 22 mature miRNAs encoded from 12 pre-miRNAs, all of which are functionally incorporated into the RISC complex [34]. This study also identified and validated two miRNAs that had not previously been detected by other methods: miR-US22 and miR-US29 [34]. Unlike other herpes viruses, which have clusters of miRNAs near important latency regulatory regions, HCMV miRNAs are scattered throughout the genome (Figure 1a) [31,33,34]. The genomic organization of the HCMV miRNAs may reflect our still-limited understanding of HCMV gene expression during latency. A second HCMV miRNA deep sequencing analysis performed by Meshesha et al. [35] confirmed many of the results from the initial deep sequencing study [34], but also identified miR-UL59 and miR-UL69 as new HCMV miRNAs. However, these miRNAs were not functionally validated in this study, nor were they identified in any other study of HCMV miRNA expression. Moreover, there are no homologues to miR-UL59 and miR-UL69 in Chimpanzee CMV (CCMV) [35], despite the otherwise high level of conservation between HCMV and CCMV miRNAs [32,36,37]. Additionally, miR-UL70-3p, a miRNA originally identified by Grey et al. [33], was not detected in either deep sequencing analyses [34,35]. Commercially designed probes for the amplification of miR-UL59, miR-UL69, and miR-UL70-3p are available; however, these miRNAs have not been functionally validated in HCMV infected samples, and studies of these miRNAs should be interpreted with caution. Finally, both deep sequencing analyses detected miR-US4, but the miRNA differed by 5 base pairs at the 5’ end compared to the originally identified miRNA [33], significantly altering the seed sequence. 

Efforts to elucidate miRNA expression during latency have yielded conflicting results, likely due to the inherent difficulties of studying HCMV latency. During natural latent infection in humans, the viral genome is maintained in 1:10,000 to 1:100,000 cells, and no means to detect or enrich for these infected cells has been developed [38]. Thus, in vitro infection systems have been used to study HCMV latency, each of which carries its own caveats [39]. The majority of studies investigating the expression of HCMV encoded miRNAs during latency have been performed in the THP-1 cell line, a human monocytic cell line derived from an acute monocytic leukemia patient [40,41,42]. HCMV infection of THP-1 cells supports a low-level persistent infection with limited viral gene expression, but reactivation in these cells is very ineffective, and so may not accurately represent a truly latent infection [18]. The studies investigating HCMV miRNA expression in THP-1 cells do not agree on which miRNAs are expressed during infection, although most reported that miRNA expression patterns differed between latent and lytic models of infection [40,41,42,43]. Two recent studies investigated miRNA expression in latently-infected primary cells which more closely reflects latent miRNA expression in vivo. Lau et al. [43] detected all HCMV miRNAs at 4 days post infection (dpi) in CD34+ HPCs and CD14+ monocytes, whereas Pan et al. [44] found that most miRNAs are expressed at very low levels at 10 dpi in CD34+ HPCs, with the exception of miR-UL22A-5p, miR-UL112-3p, and miR-UL148D. Pan et al. noted that all miRNAs were expressed at 4 dpi, consistent with findings by Lau et al. [43]. Further studies are required to uncover miRNA expression patterns during the establishment of latent HCMV infection in HPCs and during reactivation from latency.

miRNAs are encoded using minimal genetic material, yet have significant regulatory potential, as a single miRNA could regulate hundreds of cellular transcripts [45]. miRNAs therefore represent a powerful means for the virus to fine-tune expression of cellular and viral proteins, and likely have a significant role to play during latency. Below, we discuss the studies which have identified and validated targets (summarized in Table 1) of HCMV miRNAs that are involved in immune evasion, survival, and proliferation of infected cells, as well as how HCMV miRNAs play important roles in latency and reactivation. 

## 2. HCMV miRNAs and Immune Evasion

### 2.1. NFκB Signaling

Interactions between HCMV and the NFκB signaling pathway are complex and contradictory. Evidence clearly indicates that viral binding and entry trigger the activation of the NFκB signaling pathway [67,68,69,70,71]. However, several viral proteins and miRNAs block aspects of the signaling pathway, while other viral proteins can stimulate signaling through the NFκB pathway (reviewed in [72]). While on the surface it may seem perplexing that HCMV encodes proteins that both stimulate and antagonize the NFκB signaling pathway, this is likely a function of the diverse cell types that CMV infects and the disparate life cycles that occur in each cell type. 

HCMV miRNAs interfere with the NFκB signaling pathway at several key steps. HCMV miR-UL112-3p directly targets the 3’ UTR of the pattern recognition receptor TLR2 [62], which recognizes HCMV gB and gH [73,74], as well as other bacterial [75] and viral ligands [76,77]. Downregulation of TLR2 protein levels results in reduced stimulus-dependent ubiquitination of IRAK1, an adaptor molecule downstream of TLR2 in the NFκB pathway. A miR-UL112-3p mutant virus was incapable of reducing TLR2 protein levels, but this phenotype could be rescued by expression of a miR-UL112-3p mimic. Additionally, the expression of proinflammatory cytokines was reduced in miR-UL112-3p-expressing cells upon stimulation with a TLR2 agonist [62]. This data clearly demonstrates that TLR2 is a target of miR-UL112-3p during viral infection, and may have functional implications in the activation of proinflammatory cytokine signaling in response to external stimuli.

miR-UL112-3p and miR-US5-1 were each shown to target key components of the IKK complex, IKKα and IKKβ. By reducing IKKα and IKKβ protein levels, miR-US5-1 and miR-UL112-3p could prevent the degradation of IκBα and limit the production of proinflammatory cytokines upon IL-1β or tumor necrosis factor (TNF) treatment. Importantly, in this study, the authors demonstrate that infection with a miR-US5-1/miR-UL112-3p double mutant virus resulted in increased IKKα and IKKβ protein levels and increased proinflammatory cytokine secretion in human fibroblasts, endothelial cells, as well as in THP-1 cells. Infection with a virus lacking miR-US5-1 and miR-UL112-3p but containing short hairpin RNAs (shRNAs) targeting IKKα and IKKβ resulted in a return to wild type (WT) levels of proinflammatory cytokine release, indicating that the phenotype of the miR-US5-1/miR-UL112-3p mutant virus was due to the targeting of IKKα and IKKβ by these miRNAs [56]. Whether viral miRNAs act to modulate the NFκB signaling induced by viral proteins, especially during latency, is an interesting question that remains to be addressed.

### 2.2. Inhibition of NK Cell Activating Receptors

Natural killer (NK) cells are innate immune lymphocytes that play a critical role in suppressing HCMV infection in vivo [78]. NK cells survey their surroundings and receive signals from both activating and inhibitory receptors, the balance of which determines whether or not the NK cell commits to cytotoxic killing of the interacting cell. NK cell inhibitory receptors interact with both classical and non-classical major histocompatibility (MHC) class I molecules on the target cell [79]. HCMV, using proteins from the US2-11 region, downregulate MHC from the cell surface in order to prevent the recognition of viral peptides by CD8+ T cells, which leaves the cells susceptible to NK cell killing [80,81,82]. To circumvent this problem, HCMV encodes UL16, which interacts with the NK cell activating receptors ULBP1 and ULBP2 and retains them in the endoplasmic reticulum [83,84]. Additionally, HCMV miR-UL112-3p targets the NK cell activating receptor MHC class I-related chain B (MICB). While decreasing the abundance of MICB in the infected cell, miR-UL112-3p can also inhibit NK cell cytotoxicity in vitro [58]. The authors went on to determine that miRNAs from all classes of herpes viruses target MICB, indicating that MICB is a significant innate immune modulator during herpes virus infection [85]. The miR-UL112-3p binding site partially overlaps that of a cellular miRNA, theoretically impeding host regulation of the MICB transcript during viral infection while ensuring that the miRNA target site within the MICB 3’ UTR is maintained [86]. Additionally, an interesting follow-up study determined that miR-UL112-3p acts cooperatively with the cellular miRNA miR-376a for optimal downregulation of MICB expression during HCMV infection [87].

### 2.3. miRNA Regulation of Cytokine Production and Secretion

Proinflammatory and antiviral cytokines play a significant role not only in limiting lytic replication of HCMV, but are also critical for inducing cellular differentiation and viral reactivation from latency [18]. While numerous viral proteins have been shown to modulate cytokine production and secretion [88,89,90,91], in latency the viral gene expression profile is significantly reduced [92,93,94,95], and thus, viral miRNAs represent a means to modulate cytokine levels in the absence of robust viral protein expression. miR-UL148D targets the activin receptor ACVR1B in order to limit IL-6 secretion from infected monocytes [43]. The authors show that infection with a miR-UL148D mutant virus results in increased ACVR1B protein levels but no changes in latency or reactivation in a CD14+ monocyte infection model. However, the miR-UL148D mutant virus-infected monocytes secreted more IL-6 in response to activin than wild type infected cells [43]. miR-UL148D was also shown to target the RANTES transcript in order to reduce its production and release [60]. RANTES is a proinflammatory chemokine involved in recruitment of T cells, basophils, and eosinophils [96], and plays an important role in recruitment of mononuclear cells to sites of infection. Lytic infection with a miR-UL148D mutant virus results in increased transcript levels of RANTES, as well as increased secretion of the chemokine into cell culture supernatants [60]. HCMV also encodes the UL22A protein, which acts as a soluble RANTES decoy receptor and is highly expressed during lytic and latent infection [97]. Thus, HCMV encodes non-coding RNAs as well as proteins that inhibit the production and effect of RANTES on the infected cell, highlighting the importance of this chemokine during viral infection in vivo. Additionally, HCMV mIR-UL112-3p targets the 3’ UTR of IL-32, a novel cytokine involved in NFκB, AP-1, and p38MAPK signaling, and, through targeting unknown proteins, can affect Interferon (IFN) production [57]. Additionally, miR-US25-1-5p targets the 3’ UTR of CD147, a glycoprotein in the immunoglobulin superfamily that serves an antiviral role in HCMV infection [49]. Knockdown of CD147 or pre-expression of miR-US25-1-5p prior to HCMV infection results in decreased production of proinflammatory cytokines [49]. However, given that pre-expression of several HCMV miRNAs is known to interfere with virus replication [37,58] this observation should be interpreted with caution.

Perhaps the strongest evidence that viral miRNAs play an essential role in regulating the functions of cellular cytokines comes from the study by Hook et al. [63], which determined that three viral miRNAs, miR-US5-1, miR-US5-2, and miR-UL112-3p, target multiple components of the endocytic recycling pathway to limit the release of proinflammatory cytokines. The authors identified at least 5 cellular proteins (Vamp3, Rab5C, Rab11a, SNAP23 and CDC42) that function in the endocytic recycling pathway that are targets of the HCMV miRNAs. A combination of the three HCMV miRNAs was even more effective at preventing release of IL-6 and TNFα compared to small interfering RNAs (siRNAs) against the combined targets, suggesting additional targets within the pathway or additional functions of the viral miRNAs that contribute to this phenotype. Additionally, infection with a miR-US5-1/miR-US5-2/miR-UL112-3p triple miRNA mutant virus resulted in significantly increased secretion of IL-6 compared to wild type infection. Thus, miRNAs play a critical role in preventing release of cytokines as another means of regulating the effects of proinflammatory cytokines.

The miR-US5-1/miR-US5-2/miR-UL112-3p triple mutant virus also displays a striking phenotype with respect to formation of the VAC. In the absence of the three miRNAs, the virus no longer efficiently forms a VAC, and strikingly, a VAC-like structure can be formed through transfection of the three miRNAs alone, or siRNAs against their cellular targets [63]. Thus, in addition to regulating cytokine release, modulating the expression of several endocytic recycling compartment members is essential for proper VAC formation and production of the infectious virus. Additionally, miR-US33 was shown to target the SNARE protein STX3, which could play a role in the envelopment of HCMV [98]. 

## 3. HCMV miRNA Regulation of Cell Survival and Proliferation 

### 3.1. Regulation of Apoptosis by HCMV miRNAs

Modulating extrinsic or intrinsic apoptotic signals is another viral function that plays an important role during lytic and latent infection. While the anti-apoptotic functions of HCMV proteins have been extensively investigated [99,100,101,102], the role viral miRNAs play in regulating apoptosis is only beginning to be appreciated. Some evidence suggests that viral miRNAs, including miR-UL36 and miR-UL148D, block this process [55,61], while miR-US4 and miR-US25-1 may act to promote apoptosis [48,59]. A proteolytic cleavage product of p21-activated kinase 2 (PAK2) is involved in inhibition of apoptosis in response to Fas, TNFα, and UVC light, and is a target of miR-US4-5p. Expression of miR-US4-5p enhanced apoptosis induced by serum withdrawal similarly to cells expressing an siRNA against PAK2 [59]. In addition, miR-US25-1 targets the 5’ UTR of BRCC3 [50], an important protein in the DNA repair pathway. Reducing BRCC3 protein levels may be related to the ability of miR-US25-1 to enhance caspase 3 activation and apoptosis in endothelial cells in response to oxidized low density lipoprotein [48]. On the other hand, miR-UL36-5p targets SLC25A6 (ANT3), an adenine nucleotide transporter responsible for translocating ADP and ATP across the mitochondrial membrane. miR-UL36-5p inhibits apoptosis induced by serum withdrawal similar to an ANT3 siRNA [61]. Additionally, immediate early gene X-1 (IEX-1) was identified as a pro-apoptotic factor in both endothelial and epithelial cells [103,104] targeted by miR-UL148D [55]. miR-UL148D expression reduced apoptosis induced by ectopic expression of IEX-1 in 293 cells [55]. Thus, evidence exists to support both pro- and anti-apoptotic functions of HCMV miRNAs. The expression kinetics and cell type specificity of miRNA expression likely determines the outcome of miRNA modulation of apoptosis during HCMV infection.

### 3.2. Cell Cycle Regulation by HCMV miRNAs

For HCMV to regulate latency in CD34+ HPCs, maintenance of the quiescent state of the progenitor cell, as well as stimulation of differentiation upon viral reactivation, are critical functions for the virus. Therefore, modulation of the cell cycle is likely to be important for the regulation of latency and reactivation of HCMV. The first study to suggest that viral miRNAs play a role in cell cycle regulation identified numerous targets of miR-US25-1 using a RISC immunoprecipitation (RISC-IP) technique. Grey et al. [50] identified five cell-cycle-regulating genes, including cyclin E2, that have miR-US25-1 binding sites within the 5’, rather than 3’ UTRs. Cyclin E2 levels were increased in fibroblasts infected with a miR-US25-1 knockout virus compared to wild type infected cells, although this study did not describe any changes in cell cycle during infection with a miR-US25-1 mutant virus. miR-US5-1, through targeting the DNA replication inhibitor geminin, has also been implicated in controlling the cell cycle during HCMV infection. Pre-expression of miR-US5-1 or an siRNA against geminin reduced viral DNA copy numbers during infection of U373 cells, although this result should be interpreted with caution, as the effects of pre-expressing miR-US5-1 or a geminin siRNA on viral entry or gene expression was not assessed. Expression of miR-US5-1 or an siRNA targeting geminin outside the context of infection increased the number of cells that enter S phase of the cell cycle and enhanced proliferation of U373 cells, suggesting that miR-US5-1 plays a role in cell cycle regulation [54]. Finally, eIF4A1 was identified as a target of miR-US25-2-3p. Pre-expression of miR-US25-2-3p decreased both HCMV and host genomic DNA synthesis, and moderately reduced cap-dependent translation and cell proliferation; but again, these studies should be interpreted with caution, as the effects of pre-expression of miR-US25-2-3p on viral entry and gene expression were not assessed [51]. None of these studies investigated the effect of miRNA-mediated cell cycle effects during latent infection, but it is tempting to speculate that these miRNAs are important for fine-tuning the switch between latency and reactivation of the virus through their ability to regulate the proliferation and differentiation of HPCs. 

The most extensive analysis of HCMV miRNA effects on cell-cycle regulation come from the study by Pan et al. [44], which examined the role of miR-UL148D targeting immediate early response gene 5 (IER5) in HCMV latency and reactivation in CD34+ HPCs and the Kasumi-3 monocytic cell line. Through targeting IER5, miR-UL148D was able to increase CDC25B expression, which the authors show plays an important role in latency establishment. The reciprocal relationship between IER5 and CDC25B protein levels is inverted in CD34+ HPCs infected with a miR-UL148D mutant virus. In contrast to the study by Lau et al. [43], Pan et al. found that a miR-UL148D mutant virus was incapable of establishing latency in CD34+ HPCs as measured by viral transcript expression. Similar results were obtained in the Kasumi-3 model of HCMV infection, where increased viral DNA copy number and IE gene expression during miR-UL148D mutant virus infection could be rescued by the expression of a miR-UL148D agomir [44]. The authors go on to show that high levels of CDC25B are required to limit IE gene expression in Kasumi-3 cells, and link CDC25B expression to CDK-1, a cyclin dependent kinase known to regulate IE gene expression. Further studies using latency model systems will be required to fully elucidate the molecular mechanisms of cell cycle control during HCMV infection. 

## 4. HCMV miRNAs and Viral Latency

For latency to be achieved, HCMV must restrict and modulate its own gene expression in order to prevent viral replication and release. HCMV must also manipulate the cellular environment to best maintain the viral genome and support essential cellular functions while avoiding detection by the intrinsic, innate, and adaptive immune systems. Finally, the virus must remain poised to reactivate when the appropriate differentiation stimuli are encountered, while also preventing spurious reactivation due to variations in basal transcript levels or sub-optimal activation of cellular signaling pathways. From their initial discovery, herpes virus miRNAs were hypothesized to play important roles in each of these processes. Their non-immunogenic nature, along with their ability to potentially target hundreds of transcripts, make miRNAs an attractive means to modulate cellular signaling pathways during viral latency, where proteins that normally regulate cellular processes may not be expressed to sufficient levels. Much work has furthered this hypothesis in the gamma- and alpha-herpes virus fields, where well-accepted in vitro and in vivo model systems exist to study viral latency [105,106,107,108,109,110]. The study of beta-herpes virus miRNAs has been hampered by the species specificity of these viruses and the difficulties associated with in vitro latency culture systems [39]; however, progress has been made in addressing the important role of HCMV miRNAs in latency. 

In order for latency to be established in undifferentiated cells, expression of the viral trans-activating proteins must necessarily be suppressed. Therefore, it was intriguing when the HCMV trans-activating protein IE72 was identified as a target of miR-UL112-3p [37,65]. Pre-expression of miR-UL112-3p in fibroblasts was able to partially inhibit IE gene expression and reduce viral DNA copy number. Thus, Grey et al. [37] were the first to propose that targeting IE72 may play an important role in latency establishment. Shortly thereafter, these results were confirmed, and it was shown that deletion of miR-UL112-3p from the virus resulted in increased IE72 expression [65]. Further studies determined that HSV, EBV, and KSHV also use viral miRNAs to target their transcriptional trans-activators, indicating a common mechanism to limit viral gene expression during latency establishment [105,109,111,112,113,114,115,116]. In order to directly test the importance of IE72 regulation by miR-UL112-3p in latency, a virus lacking the miR-U112-3p binding site within the 3’ UTR of IE72 was generated, and IE gene expression was assessed in CD14+ monocytes. Although this virus was capable of establishing latency in the CD14+ monocyte model, the lack of regulation of IE72 by miR-UL112-3p resulted in a population-wide increase in spurious IE gene expression and increased killing of infected cells by IE-specific CD8+ cytotoxic T lymphocytes [64]. This data would suggest that miR-UL112-3p targeting of IE72, while not essential for latency establishment, plays an important role in limiting IE gene expression and protecting infected cells from the adaptive immune response in order to maintain the pool of latently-infected cells in the host. In addition to targeting IE72, other viral transcripts are targets of HCMV miRNAs, including UL112/UL113 and UL120/UL121, which are targeted by miR-UL112-3p [37], US7, targeted by miR-US5-1 and miR-US5-2 [66], and UL138, targeted by miR-UL36 [117]. Additionally, miR-UL112, miR-UL148D, and miR-US33 are encoded antisense to the open reading frames (ORFs) of UL114, UL150, and US29, respectively, while miR-UL36 is encoded within the intron of UL36 [33,34,37,58]. Expression of some of these miRNAs may lead to disruption of these transcripts through direct targeting or decreased transcript levels through DROSHA processing [33,42,58]. These interactions may play an important role in limiting viral gene expression during latency, although this remains to be experimentally validated.

At present, only two studies have investigated the role of HCMV miRNAs in latency and reactivation in primary cell types, as described above, and give conflicting results regarding the phenotype of a miRNA mutant. The discrepancy between the studies of Lau et al. [43] and Pan et al. [44] with regards to the phenotype of the miR-UL148D mutant virus has not been resolved, but could be due to the different cell types studied (CD14+ monocytes vs CD34+ HPCs and the Kasumi-3 cell line) or the viral strains used (TB40E vs the relatively uncharacterized NR-1 strain). 

In vivo systems for studying HCMV have also been difficult to develop due to the species specificity of the virus. Mouse cytomegalovirus (MCMV) is often used as a model for HCMV infection, as MCMV is able to establish latency and reactivate from multiple organs [118,119,120], and displays similar pathogenesis to HCMV [121]. MCMV expresses 21 mature miRNAs from 18 pre-miRNAs that are scattered throughout the genome (Figure 1b). Unfortunately, there is no sequence homology between MCMV and HCMV miRNAs [122], although this does not preclude targeting of similar proteins or cell signaling pathways by the different viral miRNAs. Nevertheless, several studies have investigated the role of MCMV miRNAs during in vivo infection. Mutation of miR-M23-2 and miR-m21-1, the two most highly-expressed MCMV miRNAs, resulted in a severe replication defect in the salivary glands of certain mouse strains [123]. This is particularly interesting, since the salivary glands are important sites for CMV transmission [124]. The replication defect could be partially restored by depletion of NK and CD4+ T cells, suggesting that these miRNAs are important for modulating the adaptive immune system [123]. A recent study showed that tail vein injection of two MCMV miRNA agomirs inhibited viral reactivation from mouse liver [125]. The authors concluded that these miRNAs are important for the establishment or maintenance of latency; however, experiments with miRNA mutant viruses are required to more directly address this question. Rhesus cytomegalovirus (RhCMV) has also been used as a model system for studying HCMV proteins [126,127], and homology between HCMV and RhCMV miRNAs has been identified [128]. RhCMV-encoded miR-Rh183-1 is a homologue of HCMV miR-US5-2. Similar to miR-US5-2 targeting of the viral protein US7 [66], miR-Rh183-1 targets Rh186, the RhCMV homologue to HCMV US7 [128]. Thus, the RhCMV model can be used to test the importance of CMV miRNAs in a tractable in vivo system more closely related to that of HCMV. In order to circumvent the issue of species specificity and directly assess HCMV infection in vivo, a human CD34+HPC-engrafted NOD-*scid*IL2Rγc null (huNSG) mouse model has been developed. Humanized mice infected with HCMV can support a latent viral infection that reactivates upon G-CSF-induced mobilization of stem cells to the periphery [24,129]. This mouse model has been used to interrogate the role of HCMV proteins in latency [130,131,132], and can be used to study HCMV miRNA mutant viruses in an in vivo setting.

In addition to the regulation of cellular and viral proteins using viral miRNAs, HCMV and MCMV can manipulate levels of cellular miRNAs during infection. It was originally determined that the MCMV transcript m169 contains a canonical binding site for miR-27 and acts to reduce miR-27 levels in the infected cell [133]. Additionally, a region of RNA between HCMV UL144 and UL145, termed the *miRNA decay element* (miRDE), binds miR-17 and miR-20a, and reduces their levels during lytic infection [134]. Although the functional implications of these cellular miRNA sponges have not been fully elucidated, the targeted miRNAs are involved in regulating cell cycle components, and it is tempting to suggest that this is yet another mechanism CMV utilizes to regulate the cellular environment during latency.

The importance of HCMV miRNAs in latency and reactivation is only beginning to be appreciated. Many of the HCMV miRNA targets identified during lytic infection, including components of the NFκB and apoptosis pathways, cytokines, and chemokines, as well as proteins involved in cell cycle regulation, could all hypothetically be important for latency establishment, maintenance, and reactivation. Testing functional phenotypes of miRNA mutants in primary cell latency models will be essential to uncovering the importance of the viral miRNAs in these processes.

## 5. Considerations for Studying HCMV miRNAs

One essential approach to study the relevance of HCMV miRNAs is to examine the functional phenotypes of viral miRNA mutants. Removal of a single miRNA from the HCMV genome generally has little impact on lytic replication [50,56,63,64,65]. However, the mutation of three viral miRNAs from the HCMV genome has a tremendous impact on lytic viral replication [63]. Thus, when studying viruses with multiple miRNA mutations during lytic or latent infection, any potential replication defect of the miRNA mutant virus must be assessed. Additionally, it has been shown by several groups that pre-expression of a miRNA prior to infection can have an impact on viral replication [37,58], and must be considered when miRNA pre-expression is used to complement a miRNA mutant virus.

The complex interactions of multiple HCMV miRNAs targeting multiple components of a cellular signaling pathway resulting in a given functional phenotype during viral infection requires a comprehensive means to identify all viral miRNA targets in an infected cell. Identifying the targetome of a viral miRNA is no small feat, and miRNA target identification algorithms that depend on evolutionary conservation are generally not useful for the study of viral miRNA targets [107,135]. High throughput systems have been developed to comprehensively identify miRNA targets in infected cells. High throughput sequencing of RNA isolated by crosslinking immunoprecipitation (HITS CLIP) has been performed using lytically-infected human fibroblasts as a means to identify HCMV miRNA targets temporally throughout viral infection [46]. This study identified and validated miRNA targets involved in cell cycle regulation, apoptosis, and IFN signaling, and provides a resource of potential HCMV miRNA targets, although one that requires further validation. More sensitive high-throughput methods, such as photoactivatable ribonucleoside-enhanced crosslinking and immunoprecipitation (PAR CLIP), allow for more sensitive and specific identification of miRNA binding sites [122,136,137,138], and can be applied to HCMV infected cells. These systems are incredibly valuable, but utilizing lytic infection models presents technical challenges and requires complex data analysis. Alternatively, CLIP studies using overexpression of individual miRNAs in cells lines can be utilized, but requires the validation of potential target proteins during viral replication [137]. Despite their limitations, tools such as HITS CLIP and PAR CLIP are essential to uncovering a more comprehensive view of viral miRNA targets that can be functionally validated in viral infection models. 

The identification of potential miRNA targets requires an accurate knowledge of the precise seed sequence for a given miRNA. Unfortunately, the HCMV miRNA field has been hampered by the original misidentification of the sequence for miR-US4. Several studies have been published identifying miRNA targets using the incorrect seed sequence for miR-US4, including miR-US4 targeting of ERAP to regulate peptide trimming and antigen presentation, as well as miR-US4 targeting of QARS in regulation of apoptosis [52,59]. These studies should be interpreted with caution, as the correct miR-US4 seed sequence is shifted by 5 nucleotides compared to the sequence used to identify these targets. Interestingly, ERAP was also suggested as a target of the passenger strand of miR-UL112 (miR-UL112-5p). The authors report that miR-UL112-5p targeting of ERAP regulates processing and presentation of pp65 peptides to cytotoxic T lymphocytes, and identify a naturally-occurring genetic variant that escapes regulation by miR-UL112-5p [53].

Many studies in the HCMV miRNA field have used the concept of ‘phenocopying’ to identify the functional effects of a viral miRNA. Practically, this means showing that the miRNA and an siRNA or shRNA against its target behave similarly in a given functional assay. However, the functional phenotype of a viral miRNA mutant may be attributed to any number of the potentially hundreds of cellular and viral transcripts that a particular miRNA regulates. Thus, attributing a functional phenotype to one particular miRNA-targeted protein can be difficult. One means to achieve this link is through the use of shRNA(s) against a miRNA target of interest that is expressed in the context of viral infection. If a miRNA mutant virus that expresses an shRNA against the target of interest can revert the miRNA mutant phenotype back to that observed during WT infection, this is strong evidence that the functional phenotype is due to regulation of that targeted protein. This is exemplified in the work of Hancock et al. [56], who demonstrated that targeting of IKKα and IKKβ by miR-US5-1 and miR-UL112-3p was responsible for modulating proinflammatory cytokine levels during lytic infection using this approach. Alternatively, the viral miRNA binding site within the transcript of interest could be mutated using CRISPR/Cas9 technology, and these cells could be used to show the reversion of the miRNA mutant phenotype back to that observed during wild type infection.

## 6. Conclusions and Future Perspectives

**Figure 2:**
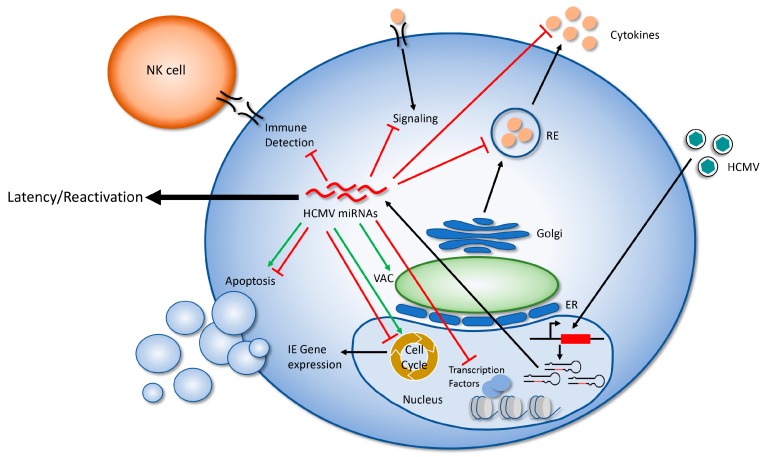
A model of HCMV miRNA regulation of the host cell. Following viral entry and translocation to the nucleus, lytic HCMV infection results in the expression of 22 mature miRNAs. These miRNAs target multiple proteins in order to modulate cellular processes including signaling, gene expression, cell cycle, apoptosis, cytokine production/secretion, formation of the virion assembly compartment (VAC), and immune detection. In this way, HCMV miRNAs create a cellular environment that supports a long term, persistent infection in the host. Red lines indicate processes that are inhibited by miRNAs; green arrows indicate processes that HCMV miRNAs promote.

The study of HCMV miRNA targets and their relevance to viral replication in a variety of cell types has progressed more slowly than for other herpes viruses, likely due to the significant RNA degradation that occurs during lytic infection [31,46] and the paucity of tractable model systems for studying the most important aspect of HCMV biology: latency and reactivation. However, the study of HCMV miRNA targets has identified some intriguing means that the virus uses to manipulate the host cell and its environment in order to avoid detection by the immune system and make the cell hospitable to long-term viral infection (Figure 2). A more complete understanding of the viral miRNA targetome will greatly expand our understanding of how HCMV manipulates the host cell during infection, and will likely uncover novel means of viral regulation. While individual miRNAs are generally thought to act as fine tuners of their target proteins, study of HCMV miRNAs reveals that these small RNAs, alone or in combination, have significant regulatory potential. Understanding the role of HCMV miRNAs in latency and reactivation in CD34+ HPCs is critically important to uncovering their most important functions, and makes understanding the targets and functions of HCMV miRNAs a worthy area of continued study.

## Figures and Tables

**Table 1:** Validated HCMV miRNA targets. List of viral and cellular targets of HCMV miRNAs that have been validated by western blot, along with any known cellular effect of the miRNA(s).

Target	miRNA	Effect of the miRNA	Reference
**ACVR1B**	miR-UL148D	Inhibits IL-6 secretion in response to activin	[43]
**ATG5**	miR-UL112-3p miR-US22-5p miR-US29-5p	Inhibits autophagy	[46]
**BclAF1**	miR-UL112	Promotes viral gene expression and replication	[47]
**BRCC3**	miR-US25-1	Promotes apoptosis	[48]
**CASP2**	miR-US4-5p	Inhibits apoptosis	[46]
**CASP3**	miR-US25-2-3p miR-UL112-5p miR-UL22A-5p	Inhibits apoptosis	[46]
**CASP7**	miR-UL22A-3p miR-US4-3p	Inhibits apoptosis	[46]
**CCND1**	miR-US33-5p	Cell cycle?	[46]
**CD147**	miR-US25-1-5p	Reduced proinflammatory cytokine production	[49]
**CDK6**	miR-UL36-3p miR-US4-3p miR-US5-1 miR-US5-2-3p miR-US25-1-3p miR-US25-2-3p	Suppress cell cycle progression	[46]
**Cyclin E2** **TRIM28**	miR-US25-1	Cell cycle?	[50]
**eIF4A1**	miR-US25-2-3p	Inhibits HCMV replication and host cell proliferation	[51]
**ERAP**	miR-US4? * miR-UL112-5p	Inhibits processing and presentation of pp65 to cytotoxic T lymphocytes	[52,53]
**FAS**	miR-UL36-3p miR-US5-1 miR-US5-2-3p	Inhibits apoptosis	[46]
**Geminin**	miR-US5-1	Enhanced cell proliferation Increased number of cells in S phase	[54]
**IER5**	miR-UL148D	Increased CDC25B expression Limit IE gene expression Latency Establishment	[44]
**IEX-1**	miR-UL148D	Inhibits apoptosis	[55]
**IKKα and IKKβ**	miR-US5-1 miR-UL112-3p	Modulate NFκB pathway Reduced proinflammatory cytokine secretion	[56]
**IL-32**	miR-UL112-3p	Reduced IL-32 expression during HCMV infection	[57]
**MICB**	miR-UL112-3p	Inhibits NK cell cytotoxicity	[58]
**PAK2**	miR-US4-5p	Promotes apoptosis	[59]
**QARS**	miR-US4? *	Inhibits apoptosis	[52]
**RANTES**	miR-UL148D	Reduced RANTES secretion	[60]
**SLC25A6 (ANT3)**	miR-UL36-5p	Inhibits apoptosis	[61]
**TLR2**	miR-UL112-3p	Reduced proinflammatory cytokine production	[62]
**Vamp3** **Rab5c** **Rab11a** **SNAP23** **CDC42**	miR-US5-1 miR-US5-2 miR-UL112	VAC Formation Inhibits proinflammatory cytokine secretion	[63]
**HCMV IE72**	miR-UL112-3p	Limit IE gene expression during latency Evade detection by CD8+ cytotoxic T lymphocytes	[64,65]
**HCMV UL112/UL113** **HCMV UL120/UL121**	miR-UL112-3p	Inhibits IE gene expression and HCMV DNA replication	[37]
**HCMV UL138**	miR-UL-36	Promotes HCMV DNA synthesis	
**HCMV US7**	miR-US5-1 miR-US5-2	Reduced US7 expression	[66]

* The correct miR-US4 seed sequence differs by 5 base pairs compared to the sequence used in these studies [34].
